# ADCC-Mediated CD56^DIM^ NK Cell Responses Are Associated with Early HBsAg Clearance in Acute HBV Infection

**DOI:** 10.20411/pai.v3i1.228

**Published:** 2018-02-19

**Authors:** Wen-Han Yu, Cormac Cosgrove, Christoph T Berger, Patrick C Cheney, Marina Krykbaeva, Arthur Y Kim, Lia Lewis-Ximenez, Georg M Lauer, Galit Alter

**Affiliations:** 1 The Ragon Institute of MGH, MIT, and Harvard, Cambridge, Massachusetts; 2 Department of Biological Engineering, Massachusetts Institute of Technology, Cambridge, Massachusetts; 3 Dept. of Biomedicine, University Hospital Basel, Switzerland; 4 Massachusetts General Hospital, Boston, Massachusetts; 5 Instituto Oswaldo Cruz, Fundação Oswaldo Cruz, Rio de Janeiro, Brazil

**Keywords:** HBV, NK cells, HBsAg, ADCC

## Abstract

**Background:**

Hepatitis B virus (HBV) affects up to 400 million people worldwide and accounts for approximately one million deaths per year from liver pathologies. Current treatment regimens are effective in suppressing viremia but usually have to be taken indefinitely, warranting research into new therapeutic approaches. Acute HBV infection in adults almost universally results in resolution of viremia, with the exception of immunocompromised persons, suggesting that the immune response can functionally cure or even eradicate HBV infection.

**Methods:**

Because immunophenotypic and functional studies have implicated a role for Natural Killer (NK) cells in HBV clearance during acute infection, we hypothesized that a distinct NK-cell profile exists in acute HBV infection that could provide information for the mechanism of HBV clearance. Using multivariate flow cytometry, we evaluated the expression of key activating and inhibitory receptors on NK cells, and their ability to respond to classic target cell lines.

**Results:**

Multivariate analysis revealed selective perturbation of the CD56,^dim^ NK-cell subset during acute infection, displaying low levels of NKp46+, NKp30+, CD160+ and CD161+ cells. Intriguingly, the CD56,^dim^ NK-cell profile predicted time to HBV surface antigen (HBsAg) clearance from the blood, and distinct NK-cell profiles predicted early (NKp30, CD94, CD161) and late clearance (KIR3DL1, CD158a, perforin, NKp46). Finally, functional analysis demonstrated that early and late clearance tracked with elevated degranulation (CD107a) or IFNγ production, respectively, in response to ADCC-mediated activation.

**Conclusion:**

The cytolytic CD56,^dim^ NK-cell subset is selectively activated in acute HBV infection and displays distinct phenotypic and functional profiles associated with efficient and early control of HBV, implicating antibody-mediated cytolytic NK-cell responses in the early control and functional cure of HBV infection.

## INTRODUCTION

Hepatitis B virus (HBV) affects up to 400 million people worldwide and accounts for approximately one million deaths per year from liver pathologies [1]. Current treatment regimens are effective in suppressing viremia but rarely result in sustained control, after cessation of therapy, when the levels of HBV surface antigen (HBsAg) become undetectable and anti-HBsAg antibodies are developed [2]. Thus, efforts to define the natural mechanisms that drive sustained virologic control may point to novel therapeutic strategies to enhance viral control in those most affected by the disease.

Acute HBV infection is marked in the peripheral blood by the appearance of HBsAg, which occurs before both the elevation of aminotransferases and other HBV proteins [3] while the disappearance of HBsAg from the bloodstream is indicative of disease resolution [4]. The vast majority of adults who contract HBV elicit robust multi-cellular responses and ultimately control infection during the acute phase [4] and remain aviremic for life. This suggests that, although HBV cccDNA might persist, a robust early immune response can provide long-term control, acting as a functional cure. However, low levels of HBV and antigen specific T-cell responses can be detected both in the liver and the blood, respectively, years after early control [5, 6], and re-emergence of HBV can occur during chemotherapy in individuals who have previously controlled HBV [7]. Thus, it is clear that the immune response plays a key role in chronic HBV control. However, the precise immunological processes that contain and prevent HBV reactivation are poorly understood, but they could provide novel insights for the development of next generation therapeutics or vaccines for the treatment of chronic HBV infection.

Natural Killer (NK) cells are key members of the innate immune system and are classically categorized into 2 main subsets by their expression of CD56 and CD16: CD56^dim^CD16^+^ (CD56^dim^) and CD56^bright^CD16^dim/-^ (CD56^bright^). These CD56^bright^ NK cells make up approximately 10% of peripheral blood NK cells, express little perforin and granzyme, but are the dominant cytokine-releasers, giving these NK cells immunoregulatory features. In contrast, CD56^dim^ NK cells constitute nearly 90% of NK cells in the blood, are thought to be more mature, and are high in perforin and granzyme content, making them the dominant cytolytic subset [8]. NK cells are candidates for immune modulation strategies aimed at HBV infection because of their enrichment in the liver [9], the site of viral replication, and their ability to elicit potent anti-viral responses early during viral infection [10]. Moreover, NK cells are activated in acute HBV infection [11, 12], can suppress viral replication in mice models of HBV [13, 14] and are important regulators of HBV-specific CD8+ T-cell responses [15], suggesting that early NK-cell responses may play a key role in the resolution of HBV infection. However, the mechanism of NK-cell control of HBV remains elusive and hence, further work is required to understand the anti-viral role of NK cells in acute infection.

Regulation of NK-cell activation involves the combination of activating and inhibitory signals mediated through distinct families of cell surface receptors including the natural cytotoxicity receptors (NCR), C-type lectins, killer-cell immunoglobulin-like receptors (KIR), NKG2 receptors, and CD16 (FcRγIII), which is the key receptor required in the recruitment of NK cells for antibody-dependent cellular cytotoxicity (ADCC). The recognition of ligands expressed on healthy, virally infected, and cancerous cells dictates the NK-cell response [16]. While NK-cell activity is predominantly inhibited in a healthy individual, NK cells are skewed toward activation in viral infection, through the combination of attenuation of inhibitory signals and presence of a strong activating signal [17].

Here, we use multivariate approaches to examine the NK cell immunophenotype and functional response in acute HBV infection. We demonstrate that acute HBV infection perturbs the more mature/cytolytic CD56^dim^ but not the CD56^bright^ NK-cell profile, and that furthermore, early clearance of HBsAg can be predicted by a distinct CD56^dim^ NK-cell phenotypic profile. Finally, we identify a unique functional signature linked to early clearance of HBV that implicates a role for ADCC-mediated cytolytic NK-cell responses in the early containment and resolution of HBV infection.

## MATERIALS AND METHODS

### Cohort

HBV-infected individuals (n = 18) were recruited at the Oswaldo Cruz Institute, Rio de Janeiro, Brazil. Individuals presented with acute symptoms of hepatitis, at less than 1 month after jaundice, and a diagnosis of acute HBV infection was confirmed by the presence of anti-HBc IgM antibodies (AxSYM CORE-M; Abbott, Delkenheim, Germany). Aminotransferases, serum HBsAg, HBeAg and total anti-HBc, anti-HBeAg and anti-HBsAg antibodies were monitored in the acute phase and were also monitored regularly thereafter until clearance. Patients were uniformly synchronized based on jaundice symptoms, for comparability. All HBV+ individuals cleared infection and were negative for HCV and HIV throughout the duration of the study. Clinical data are presented in Table 1. Healthy controls (n = 13) were recruited at Massachusetts General Hospital, Boston.

**Table 1.** Clinical data for HBV+ individuals

Clinical characteristic	Measurement
Age (years)	36 (30.75, 40.25)
Sex	70.6 % Male
	29.4 % Female
Time since jaundice (days)	19 (14, 22)
ALT (IU/ml)	598.5 (260, 985)
AST (IU/ml)	244 (154.5, 735)
Serum HBV proteins (IU/ml)	
HBsAg	271.8 (240, 329.4)
	16/16 detectable HBsAg (2 UD)
HBeAg	0.64 (0.305, 6.205)
	7/16 with detectable HBeAg (2UD)
Serum antibodies (mIU/ml)	
Anti-HBcAg IgM	3.97 (2.92, 14)
	14/14 with anti-HBcAg IgM (4 UD)
Anti-HBsAg IgG	0.2 (0, 5)
	9/16 with anti-HBsAg (2 UD)
Anti-HBeAg IgG	0.238 (0.141, 1.03)
	4/15 with anti-HBeAg (3 UD)

UD = undetermined; IU/mL = International units per milliliter of blood; mIU/mL = milli-international units per milliliter blood

### Ethics statement

All participants in this study provided written informed consent prior to sampling. The study was conducted according to the principles expressed in the Declaration of Helsinki, and was approved by the Partners Human Research Committee (Protocol #1999-P-004983/54; MGH #90-7246) and the ethics board of the Oswaldo Cruz Institute.

### NK-cell immunophenotyping

Cryopreserved peripheral blood mononuclear cells (PBMC) were stained with combinations of antibodies that comprehensively interrogate the breadth of NK-cell receptors including the killer immunoglobulin receptors (KIRs), the c-type lectin receptors (NKG2), and the natural cytotoxicity receptors (NCRs). These included CD158a-PerCP-Cy5.5 (eBioscience), KIR3DL1-Alexafluor700 (Biolegend), NKG2A-PE (Beckman Coulter), NKG2D-APC, CD94-FITC, 2B4-FITC, NKp46-PE, NKp30-APC, CD158b-FITC (all BD Biosciences), TRAIL-PE (Biolegend), CD161-FITC (Miltenyi) and CD160-Alexafluor647 (Biolegend). Dead cells were excluded using the Fixable Blue Dead Cell Stain (Life Technologies) prior to surface staining and fixation. For perforin staining, samples were then permeabilized (Perm A/B, Caltag) and stained with anti-Perforin-PerCP-Cy5.5 (eBioscience). NK cells were identified as CD3 negative lymphocytes expressing CD56 and/or CD16. At least 1500 NK cells were acquired per sample. Fluorescence minus one was used to set gates. The data were analyzed with Flowjo v9.5.4 (Treestar).

### NK cell functional profiling

NK-cell responses to target cell lines were determined as previously described [18]. PBMC were incubated with culture media alone or with K562 cells, 221 cells or antibody-coated p815 cells at an effector to target ratio of 10:1 for 5 hours in the presence of anti-CD107a PE-Cy5 (BD Biosciences, 10 ml/mL), brefeldin A (2.5 mg/mL) and Golgistop (2.5 mg/mL, BD Bioscience). Cells were then washed, stained with the Fixable Blue Dead Cell Stain prior to staining for surface receptors, then fixed, permeabilized (BD Fix/perm, BD Biosciences) and stained with anti-IFNγ-FITC, anti-MIP1β-PE and anti-TNF-Alexafluor700 (all BD Biosciences). Gates were set on media-only controls, which were also used for background subtraction prior to analysis.

### Statistical analysis

Descriptive measures (such as frequency, mean, median, standard deviation, and interquartile range) were used to summarize data. The Mann-Whitney test was used for the comparison of phenotype and functional data between 2 groups. For multivariate analyses, the NK-cell marker datasets were initially normalized using z-scores. Three multivariate approaches were then used to analyze the data including principal components analysis (PCA) (unsupervised classification), partial least-squares discriminant analysis (PLSDA) (supervised classification), and random frog analysis, all poised to define multivariate data behavior using big-data in small-to-large sample sets. The PCA analysis considered all NK-cell phenotypic markers (X) as predictors, generating novel predictor variables (principal components) as linear combinations of the original predictor variables aimed at explaining most of the variance in the dataset (X). In contrast, PLSDA models covariance between input data (X) and the response (Y) (groups are split by early/late clearance), aimed at finding novel predictor variables that explain the maximum variance in the responses (Y). However, because many variables are correlated to one another and can cause data over-fitting, Elastic-Net/Lasso was further applied prior to PLSDA to select a minimum set of variables that account for most of the variance between the groups (X), by applying sparsity constraints. A ten-fold cross-validation approach was then used to evaluate the robustness of the model classification accuracy. Additionally, to predict the time to clearance given NK-cell phenotypic markers, a partial least squares regression (PLSR) model was used, which is a version of PLSDA, but aimed at regressing all features to the continuous outcome (Y). The fitness of the model was then determined by mean-squared errors between the estimated and the observed values, and model overfitting was then estimated by cross-validation. Finally, to select the markers as the determinists that most accurately predict HBsAg clearance, an optimization algorithm modified from the random frog method [19] was used. Briefly, the algorithm randomly generated a set of selected markers in each iteration and evaluated that model by mean squared errors from 10-fold cross-validation. Five thousand iterations were performed to calculate the probabilities of each maker being selected during the optimization process. To determine the nominal *P*-value of each marker, a background model that permuted the marker profiles was used to estimate the probability distribution of randomness. All *P* values are 2-sided and *P* values of < 0.05 were considered significant. Statistical analyses were conducted using GraphPad Prism (GraphPad Prism Software, La Jolla, CA), jmp11 (SAS) or Matlab.

## RESULTS

### Acute HBV infection elicits a distinct NK-cell profile in the CD56*^dim^* population

NK-cell anti-viral responses are regulated by the interaction of multiple activating and inhibitory receptors with their ligands on virally infected cells. During viral infection, NK-cell activity is skewed both by the loss of inhibitory signals and the presence of a strong activating signal, provided through the synergistic interaction of multiple receptors [17]. Thus, successful NK-cell activation likely results from subtle changes in several receptor groups. While traditional univariate approaches may fail to fully capture such changes, multivariate analyses such as PCA can simultaneously examine changes in multiple NK-cell markers, and therefore provide greater resolution to detect changes in NK-cell profiles. Therefore, we used multivariate analyses to carefully examine the phenotypically and functionally distinct CD56^dim^ and CD56^bright^ NK-cell profiles [8] in groups of healthy and acutely infected HBV+ individuals. We integrated receptor expression profiles from the killer immunoglobulin receptors (KIRs), the c-type lectin receptors (NKG2), and the natural cytotoxicity receptors (NCRs), and NK-cell function following stimulation with a panel of NK-cell targets, with the goal of identifying key NK-cell profiles that could provide evidence towards the anti-viral mechanisms of NK-cell-mediated control of HBV.

**Figure 1.**
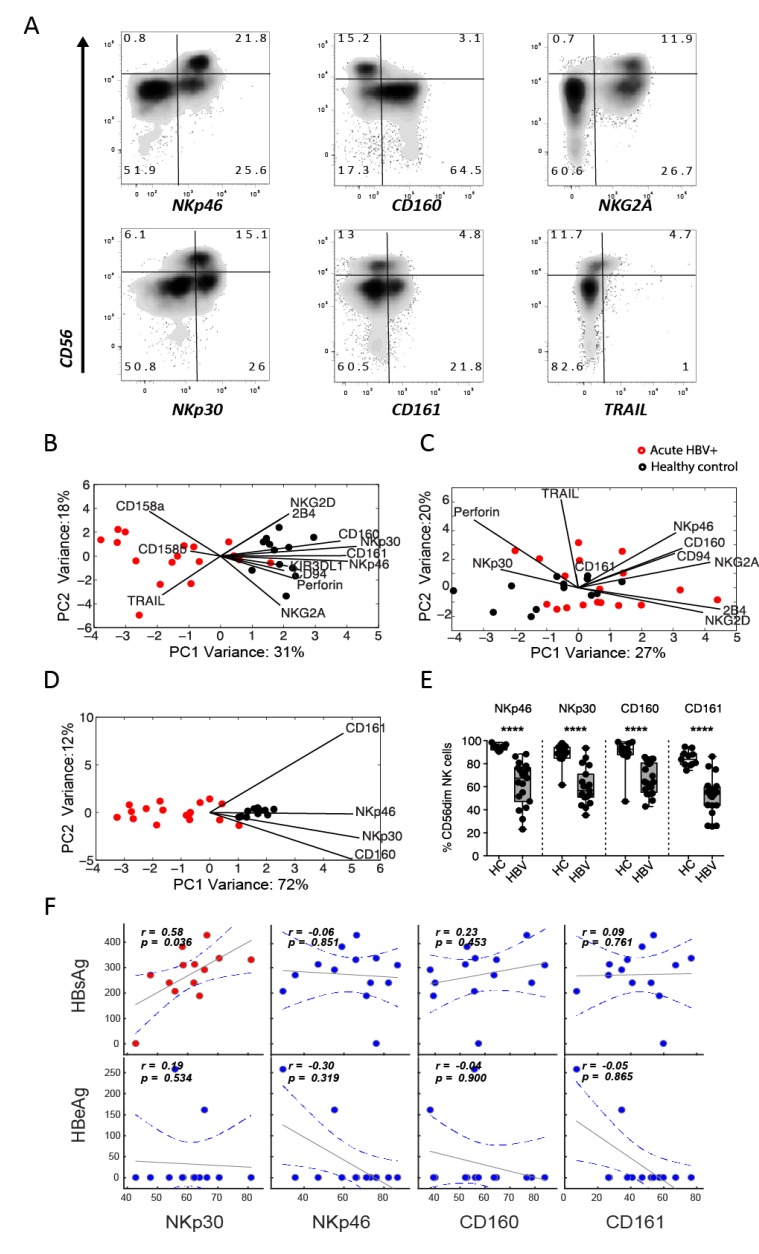
**Distinct NK-cell profiles exist among healthy controls and HBV-infected individuals.** (A) Flow plots depict the expression of a number of candidate receptors on NK-cell populations in a representative candidate in the cohort, displaying discrete separation in CD3-CD56+CD16+/-NK cells. (B-C) The score plots of the principle component analysis for CD56^dim^ NK cells (B) and CD56^bright^ NK cells (C) from both healthy controls (black) and HBV-infected individuals (red) were built based on all immunopheno-typing features. The contribution of the features to group separation were visualized by the lines in the plots, where the length of the line is proportional to how important that feature is to pulling data in its direction. (D) To further avoid over-fitting, caused by correlated changes in NK-cell markers, a second PCA was performed on CD56^dim^ NK cells using the most significantly altered features. (E) Pairwise comparison of the 4 top features, on CD56^dim^ NK cells, were compared in a univariate analysis. (F) Finally, spearman correlations between HBsAg/HBeAg levels and NKp30, NKp46, CD160, and CD161 levels on CD56dim NK cells show the relationship between these markers and HBV antigen load in HBV-infected individuals.

First, we investigated whether acute HBV infection has an impact on the NK-cell immunopheno-type. An array of NK-cell markers were profiled among CD56^dim^ and CD56^bright^ NK cells among groups of acutely infected HBV+ individuals (n = 18) and healthy controls (n = 13) (Figure 1A). Because these receptors are expressed at variable levels, we elected to use PCA to examine the multivariate distribution of these receptors. While we observed no differences in the frequency of CD56^bright^ and CD56^dim^ NK cells as a percentage of total NK cells or total lymphocytes between the groups, the NK-cell immunophenotype separated HBV+ and healthy individuals by the more mature/cytolytic CD56^dim^ (Figure 1B), but not in the CD56^bright^ subset (Figure 1C). To identify which combination of receptors best separate the 2 groups, we performed variable marker selection on the CD56^dim^ NK-cell subset. Interestingly, the best separation between HBV+ individuals and healthy controls was observed when using only 4 NK-cell markers; the activating receptors NKp30, NKp46, and CD160, and the inhibitory receptor CD161 (Figure 1D). There were significantly lower frequencies of NKp30+, NKp46+, CD160+, and CD161+ CD56dim NK cells in individuals with acute HBV infection versus healthy controls (*P* < 0.001 for all, Figure 1E), demonstrating that the CD56dim NK-cell profile displays a CD160l^ow^CD161^low^NKp30^low^NKp46^low^ immunophenotype in acute HBV infection. Expression of these markers was not associated with liver inflammation (as measured by aminotransferases) or the level of HBsAg or HBV e antigen (HBeAg) in the blood, with the exception of Nkp30 (Figure 1F). Specifically, despite an overall decline in NKp30 levels, the levels of NKp30 correlated with HBsAg levels pointing to an indirect link between this natural cytotoxicity receptor and viral clearance. Thus, similar to previous reports of acute HCV resolution [20], loss of NKp30 may be a marker of more effectively or recently responsive NK cells, further pointing to a role for NK cells in the antiviral response to HBV. Together, the data demonstrates that the CD56^dim^ NK-cell subset but not the CD56^bright^ subset is significantly perturbed in the peripheral blood during acute HBV infection, driven by the down-regulation of a limited set of activating and inhibitory receptors.

### A unique CD56*^dim^* NK-cell profile in acute infection predicts early clearance of HBsAg

**Figure 2.**
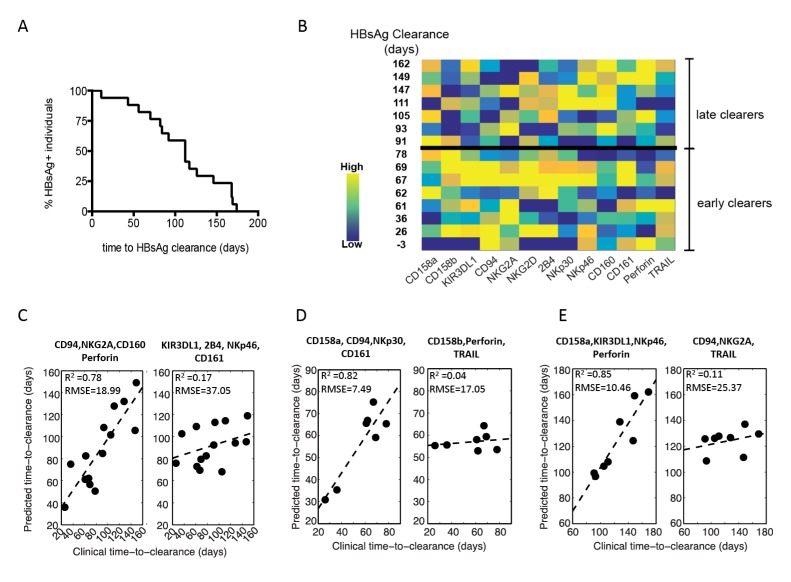
**Predictions of time-to-HBV clearance using NK-cell profiles.** (A) A Kaplan–Meier survival curve depicts the time to HBsAg clearance across all HBV-infected individuals, demonstrating a broad range of clearance rates. (B) The acute CD56^dim^ NK-cell marker profile with the corresponding time to clearance of HBsAg. (C-E) A PLSR-predicted time-to-clearance, after feature reduction, was performed (C) across the complete HBV+ cohort, (D) early clearers and (E) late clearers (early versus late were separated by the median time-to-clearance, 83 days). For each group the robustness of model prediction, presented as R-squared and root-mean-square-error (RMSE), was evaluated between the best set of the variables (left panel) versus randomly selected variables (right panel).

HBsAg is one of the earliest HBV proteins detected in the blood during HBV infection, and clearance of HBsAg from the blood correlates with disease resolution [4]. Interestingly, we observed that time to HBsAg clearance was highly variable within our cohort (Figure 2A), suggesting that some individuals were capable of clearing infection more efficiently than others. Given that we observed a skewed CD56^dim^ NK cell phenotype in acute HBV infection, and that NK cells have previously been implicated in control of HBV infection [11, 13, 14], we asked if there was a relationship between the acute CD56^dim^ NK-cell profile (Figure 2B) and time to clearance of HBsAg (Figure 2A). To answer this, we combined partial least-squares regression (PLSR) with variable selection to create a model to predict time to clearance, based on the CD56^dim^ NK-cell profile. We next compared the predicted time to clearance with the actual time to clearance measured clinically, with the r-squared value indicating the accuracy of the model. The model demonstrated that a key subset of NK-cell markers was important for predicting clearance. Specifically, when including all individuals, the best model for predicting time to clearance was one trained using a combination of CD94, NKG2A, CD160 and perforin (R^2^ = 0.78127, RMSE = 18.99; Figure 2C), while a model trained using 4 randomly selected markers could not predict time to clearance (R^2^ = 0.16731, RMSE = 37.05; Figure 2C). Moreover, deeper inspection of these markers on CD56^dim^ NK cells with respect to time to clearance, further highlighted the robust direct relationship between CD94, NKG2A, and CD160 and time to clearance (Figure 2B). These data suggest that a distinct subset of NK cells may be important in the clearance of HBsAg during acute HBV infection.

NK cells are members of the innate immune system and can act early during acute infection as they do not require prior antigen sensitization. Indeed, NK-cell responses peak prior to those of T cells during acute HBV infection and before the disease is cleared [11]. Thus, it is plausible that the individuals who clear the disease earliest in our cohort may do so by a more innate, NK-cell-mediated mechanism. We therefore hypothesized that a distinct predictive NK-cell profile associated with HBsAg clearance would exist in individuals who clear early compared to those who clear later. To test this, we stratified our cohort into early and late clearers based on the median time to HBsAg clearance (83 days). Strikingly, PLSR analysis revealed distinct profiles that predicted clearance in the 2 groups, where the model that best predicted clearance in the early clearance group was obtained using CD158a, CD94, NKp30 and CD161 (R^2^ = 0.82, RMSE = 7.49, Figure 2D), while the combination of CD158a, KIR3DL1, NKp46 and perforin best predicted time to HBsAg clearance in the late clearance group (R^2^ = 0.85, RMSE = 10.46, Figure 2E). Correlations between predicted and actual time to HBsAg clearance in models based on randomly selected variables for the early and late clearance groups were not significant (R^2^ = 0.04, RMSE = 17.05 for early clearance, R^2^ = 0.11, RMSE = 25.37 for late clearance, Figure 2D, E). These data demonstrate that there are distinct CD56^dim^ NK-cell profiles associated with early and late clearance of HBsAg, namely CD158a/CD94/NKp30/CD161 and CD158a/KIR3DL1/NKp46/perforin, respectively (Table 2).

**Table 2.** CD56^dim^ NK-cell phenotype alteration in acute HBV and association with HBsAg Clearance

	Associated with time to clearance	Associated with early clearance	Associated with late clearance
CD158a		*	*
KIR3DL1			*
NKG2A	*		
NKp46			*
CD158b			
CD161		*	
Perforin	*		*
NKG2D			
CD94	*	*	
2B4			
NKp30		*	
TRAIL			
CD160	*		

### Cytolytic ADCC-mediated responses by CD56*^dim^* NK cells are associated with early HBsAg clearance

**Figure 3.**
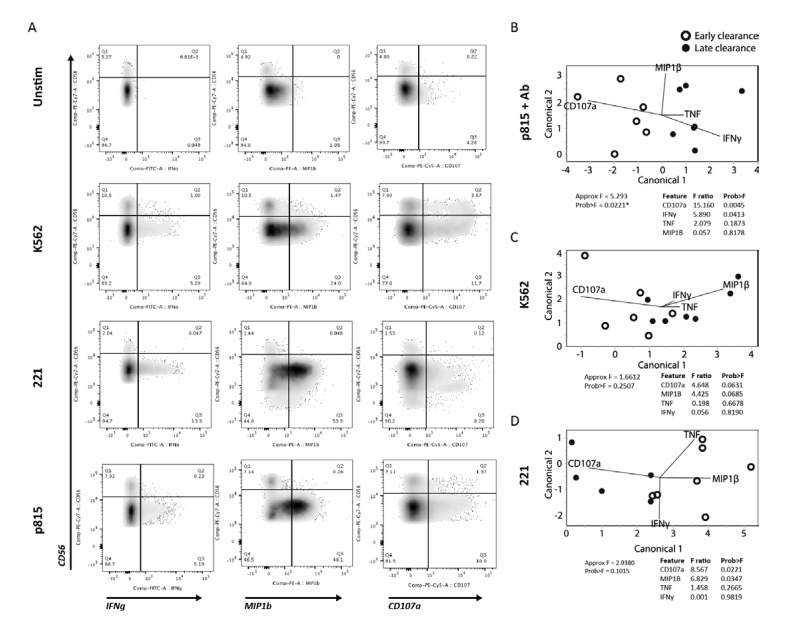
**NK-cell degranulation as a correlate of HBsAg clearance.** NK-cell activity was assessed following NK-cell stimulation with antibody-opsonized target cells to drive antibody dependent cellular cytotoxicity; ADCC (antibody+p815 cells), NKG2D activation (K562), or NCR activation (221). (A) Flow plots show the level of degranulation by CD107a, and cytokine (IFNγ) and chemokine (MIP1β) production on NK-cell populations in 3 stimulated as well as unstimulated conditions. (B-D) A multivariate PLSDA was used to differentiate the profiles of the early and late clearers activated via (B) ADCC, (C) NKG2D, and (D) NCR, distributed in the first 2-dimensional principle components, based on 4 measured features (CD107a, IFNγ, TNF, and MIP1β). The contribution of the features to group separation were visualized by the lines in the plots, and its F ratio and *P*-values were listed on the right side based on Approximate F tests.

Anti-viral NK-cell responses are mediated both through the rapid lysis of infected cells and the production of anti-viral cytokines and chemokines. We therefore asked whether the distinct receptor profile observed in early HBsAg clearance was also associated with stronger NK-cell functional responses. To answer this we examined degranulation, measured by CD107a, as well as cytokine (IFNγ, TNF) and chemokine (MIP1β) production in response to stimulation through classic NK-cell pathways; NKG2D (K562 cell line), NCR (221 cell line) or ADCC (antibody-coated p815 cells) (Figure 3A). Using partial least squares discriminant analysis (PLSDA), we compared functional responses in the CD56^dim^ NK-cell subset between early and late HBsAg clearers. PLSDA revealed distinct functional profiles in early and late clearers in response to antibody-coated p815 cells (*P* = 0.0221, Figure 3B), but not K562 (Figure 3C) or 221 cells (Figure 3D), and leave-one-out validation demonstrated 100% prediction accuracy. This data implicates ADCC-mediated NK-cell function as a key mechanism in early clearance, rather than activation through either the NKG2D- or NCR-mediated pathways. Interestingly, the key features separating the groups showed that early clearance was associated with higher CD107a (*P* = 0.0045), while conversely, later clearance was associated with elevated IFNγ (*P* = 0.0413). This perhaps suggests that ADCC-mediated CD56^dim^ NK-cell killing of infected hepatocytes is a more effective mechanism of anti-viral control in acute HBV infection than the production of non-cytopathic anti-viral cytokines.

Overall, our data demonstrate that acute HBV infection perturbs the CD56^dim^, but not the CD56^bright^ NK-cell profile suggesting that the more mature cytolytic NK-cell subset in the peripheral blood is preferentially activated during early infection. We further show that there is a distinct immunophenotypic and functional CD56^dim^ NK-cell profile that can predict early clearance of HBsAg, which implicates KIR-independent ADCC-mediated killing as a key mechanism in the control of HBV infection.

## DISCUSSION

NK cells are key members of the innate immune system and are important for the early control of viral infection. Their enrichment in the liver, the site of HBV replication, makes them ideal candidates for immunoregulatory therapies. While there is accumulating evidence for a role of NK cells in the containment and clearance of HBV [11, 13–15], the mechanism for viral control remains elusive. Here we used a cohort of acutely infected HBV+ individuals with longitudinal clinical follow-up to examine the NK-cell anti-viral response in early HBV infection. Using multivariate approaches, we observed HBV-mediated perturbations in the more mature/cytolytic CD56^dim^ NK-cell immunophenotype, which were driven by a key group of activating and inhibitory receptors. We further demonstrated that individuals who clear HBsAg early display a unique phenotypic and functional signature in acute infection that implicates antibody-mediated cytolytic NK-cell responses in the immune control of HBV.

The antiviral NK-cell response is controlled by a complex activating and inhibitory receptor repertoire that is often altered during viral infection [21]. Because of the remarkable heterogeneity and because the majority of studies have focused on single receptor dynamics, we hypothesized that a multivariate approach could reveal novel NK-cell patterns that might provide insight into the mechanism of HBV control. Interestingly, an unsupervised analysis showed that the combined expression of 4 key receptors on the CD56^dim^, but not the CD56^bright^ NK-cell subset, was sufficient to separate healthy and acutely infected individuals, namely the activating receptors NKp30, NKp46 and CD160, and the inhibitory receptor CD161. Downregulation of particular NK-cell receptors, including the aforementioned NKp30, NKp46 and CD160, generally occurs during NK-cell activation [20, 22] through the action of metalloproteases [23], suggesting selective activation of the more mature/cytolytic CD56^dim^ NK-cell subset in the peripheral blood during acute HBV infection. Along these lines, the direct correlation between NKp30 levels and HBsAg levels points to a potentially direct role for this NCR in HBV control, where individuals with the lowest levels of NKp30 exhibited the most effective clearance (Figure 1F). However, the contemporaneous downregulation of the inhibitory CD161 molecule may additionally reduce the activation threshold, shifting the balance of activation/inhibition and allowing for more effective and rapid activation of NK cells via NCRs or other NK-cell receptors which are key to the response to viral signals. Whether these changes relate to the emergence of a novel subset of NCRloCD161lo NK cells poised to respond to infection or are due to a transient loss of specific NK-cell receptors on existing activated NK-cell populations is unclear, but represent 2 potential hypotheses to explain beneficial changes in NK cells that appear to occur across infections. Moreover, previous work has demonstrated that activation of NK cells in mouse models of HBV infection was able to inhibit viral replication [13, 14], and that impaired CD56^dim^ NK-cell responses are associated with HBV persistence [24]. Thus, considering the traditionally more cytotoxic role of CD56^dim^ compared with the CD56^bright^ NK cells, these data suggest that a more direct cytolytic NK-cell response, mediated by CD56^dim^ NK cells rather than an immunoregulatory response in early HBV infection, may be central to HBV control.

The importance of the CD56^dim^ NK-cell response was underlined by PLSR analysis which demonstrated that a distinct immunophenotypic profile within this subset could predict time to HBsAg clearance [25]. This points to a potential mechanistic role for NK cells in early HBV control. Importantly, in those individuals that cleared HBsAg early, when innate immune mechanisms are likely to dominate the immune response, a unique set of NK-cell receptors could predict imminent clearance, namely the c-type lectin CD94, which forms either inhibitory or activating heterodimers with NKG2 molecules, the activating NCR NKp30, and the inhibitory c-type lectin CD161. These receptors identify a distinct NK-cell phenotypic profile in acute disease that may be linked to a more potent anti-viral innate response. While alterations in the expression of NKp30 and CD161 in acute HBV infection have been described previously [26, 27], the combined expression of this group of receptors so robustly predicted viral control in this cohort that it indicates these NK-cell receptors as potential targets for harnessing an effective and efficient innate immune response to HBV. In contrast, late clearance was best predicted by the expression of the HLA-B and HLA-C binding KIRs, KIR3DL1 and CD158a, (KIR2DL1/S1/S3/S5) as well as the activating NCR NKp46 and the cytolytic molecule perforin, suggesting that early control could occur via KIR-independent mechanisms. Thus, it is plausible that activation of NK cells through distinct pathways might result in different rates of disease control through distinct antiviral mechanisms.

Consistent with this idea, patients with early and late clearance exhibited distinct NK-cell responses (cytokines versus degranulation) to antibody-opsonized targets, suggesting that antibody-mediated recruitment of NK cells may play a key mechanism for early control of HBV infection. This is in line with the activated phenotype observed in the CD56^dim^ population, the key subset involved in ADCC-mediated killing [8]. Importantly, antibody-mediated activation of NK cells in early clearers was skewed towards elevated degranulation and away from cytokine production, while the opposite was true in late clearers, suggesting that early cytolytic ADCC responses may represent a more efficient mechanism of viral control in HBV infection. A strong early cytolytic response can impact the size of the viral reservoir in the liver, potentially allowing for better immune control of HBV [14]; CD56^dim^ NK cells make up approximately 50% of hepatic NK cells [8, 28]. Therefore, it is plausible that during acute infection, antibodies that recognize HBV proteins expressed on infected hepatocytes [29, 30] or HBV virions budding from infected hepatocytes recruit circulating NK cells that are functionally tuned for cytotoxicity, providing a highly focused mechanism for the deletion of HBV-infected hepatocytes and accelerating clearance of infection.

Thus collectively, the data reported here demonstrate striking multivariate differences in NK-cell phenotype and function that predict early and late clearance of HBV infection, implicating distinct NK-cell populations in antiviral immunity. Previous studies in chronic HCV infection point to limited changes in NK-cell populations in the liver [18]. While chronic infection does not appear to provide signals of particular NK-cell redistributions that could be responsive to infection, it is still possible that changes may occur in early disease. In particular, changes may occur in the liver that could provide insights into whether the NKp46^high^NKp30^high^ cells may be escaping into the tissues, or whether the same changes may occur in the liver in response to infection. Thus, future liver studies in early HBV infection, at a time when the adaptive immune response is just developing, could highlight changes that occur at the site of viral infection providing increased resolution of the NK-cell populations that may contribute to viral control. Yet, the involvement of antibody-mediated NK-cell activity in the early response to HBV infection points to a potentially novel route by which these cytolytic innate immune effectors may be used in a targeted manner to detect and eliminate infected cells. However, whether antibodies may be actively leveraged following therapeutic vaccination or in the form of a monoclonal therapeutic to direct elimination of infected cells is unknown. Hence, with the large number of NK cells that reside within the liver, next generation strategies aimed at directing these cells to destroy chronically infected hepatocytes could represent a new strategy to accelerate HBV cure.
